# Pneumomediastinum, Subcutaneous Emphysema, and Tracheal Tear in the Early Postoperative Period of Spinal Surgery in a Paraplegic Achondroplastic Dwarf

**DOI:** 10.1155/2013/987578

**Published:** 2013-12-18

**Authors:** Sinan Kahraman, Meriç Enercan, Özkan Demirhan, Türker Şengül, Levent Dalar, Azmi Hamzaoğlu

**Affiliations:** ^1^Istanbul Spine Center, Istanbul Florence Nightingale Hospital, 34387 Istanbul, Turkey; ^2^Department of Thoracic Surgery, School of Medicine, Istanbul Bilim University, 34387 Istanbul, Turkey; ^3^Department of Critical Care, School of Medicine, Istanbul Bilim University, 34387 Istanbul, Turkey; ^4^Department of Pulmonary Medicine, School of Medicine, Istanbul Bilim University, 34387 Istanbul, Turkey

## Abstract

Achondroplasia was first described in 1878 and is the most common form of human skeletal dysplasia. Spinal manifestations include thoracolumbar kyphosis, foramen magnum, and spinal stenosis. Progressive kyphosis can result in spinal cord compression and paraplegia due to the reduced size of spinal canal. The deficits are typically progressive, presenting as an insidious onset of paresthesia, followed by the inability to walk and then by urinary incontinence. Paraplegia can be the result of direct pressure on the cord by bone or the injury to the anterior spinal vessels by a protruding bone. Surgical treatment consists of posterior instrumentation, fusion with total wide laminectomy at stenosis levels, and anterior interbody support. Pedicle screws are preferred for spinal instrumentation because wires and hooks may induce spinal cord injury due to the narrow spinal canal. Pedicle lengths are significantly shorter, and 20–25 mm long screws are appropriate for lower thoracic and lumbar pedicles in adult achondroplastic There is no information about the appropriate length of screws for the upper thoracic pedicles. Tracheal injury due to inappropriate pedicle screw length is a rare complication. We report an extremely rare case of tracheal tear due to posterior instrumentation and its management in the early postoperative period.

## 1. Introduction

Achondroplasia was first described in 1878 and is the most common form of human skeletal dysplasia with an estimated frequency between 1 in 15,000 and 40,000 live births [1]. It's clinical manifestations include rhizomelic short stature, trident hand, genu varum, and elbow contractures. Spinal manifestations of achondroplasia include thoracolumbar kyphosis, foramen magnum stenosis, and spinal stenosis. Progressive kyphosis can result in spinal cord compression and paraplegia due to the reduced size of the achondroplastic spinal canal. The degree of kyphosis required to produce neurologic complications is not known [2]. The deficits are typically progressive, presenting as an insidious onset of paresthesia, sciatic pain, and back pain, followed by the inability to walk and then by urinary incontinence [3]. Paraplegia can be the result of direct pressure on the cord by bone or the injury to the anterior spinal vessels by a protruding bone [3]. Surgical treatment consists of posterior instrumentation, fusion with total wide laminectomy at stenosis levels, and anterior interbody support. Pedicle screws are preferred for spinal instrumentation because wires and hooks may induce spinal cord injury due to the narrow spinal canal. Pedicle lengths are significantly shorter, and 20–25 mm long screws are appropriate for lower thoracic and lumbar pedicles in adult achondroplastics [4]. There is no satisfactory scientific data about the appropriate length of screws for the upper thoracic pedicles. Tracheal injury due to inappropriate pedicle screw length is a rare complication, both in achondroplastic dwarfs or normal individuals. We report an extremely rare case of tracheal tear due to posterior instrumentation and its management in the early postoperative period.

## 2. A Case Report

**Figure 1:**
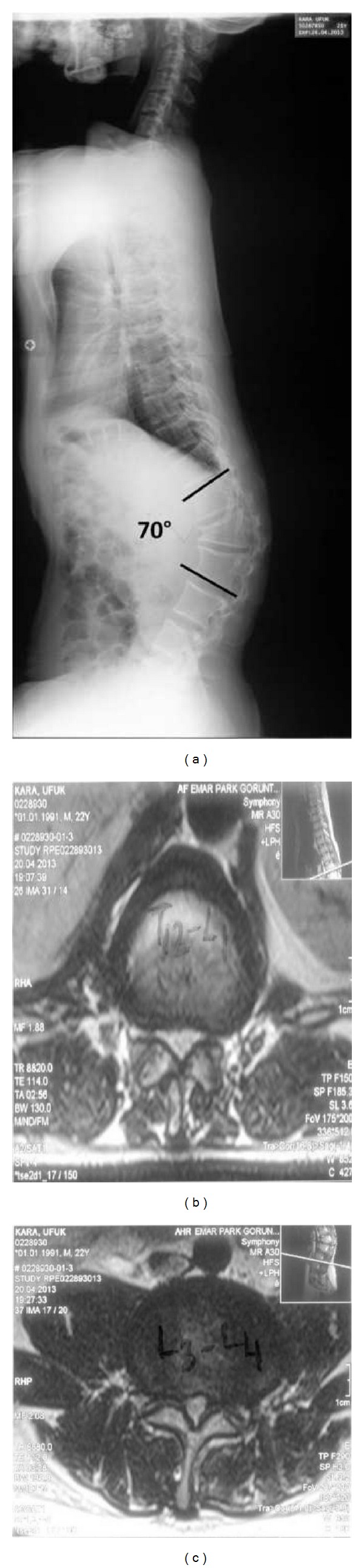
(a) Full spine X-ray showed an angular kyphosis with 70 degrees at the thoracolumbar area; (b)-(c) MRI studies showed a significant stenosis between levels T11 and L5 (MRI: magnetic resonance imaging).

A 22-year-old male achondroplastic dwarf was admitted to our center suffering from the weakness on lower extremities and paresis incomplete paraplegia. Fifteen days ago, he noted a weakness in his left leg and then fell. His neurological examination upon admission showed complete motor and sensory deficits in the left lower extremity. Motor examination of the right lower extremity showed 2/5 strength in the iliopsoas, rectus femoris, tibialis anterior, and extensor hallucis longus muscles and no sensation in the lower extremity. He had no control of bladder and bowel function. His preoperative full spine X-ray showed an angular kyphosis with 70 degrees at the thoracolumbar area and the apex at L1 level. MRI studies showed a significant stenosis between levels T11 and L5 (Figures 1(a), 1(b), and 1(c)).

After hospitalization the patient was initially given methylprednisolone, 30 mg/kg for 45 minutes, and later 5.4 mg/kg for 24 hours. There was no improvement in his neurological status after steroid therapy.

*Surgical Technique*. The surgery consisted of two stages. In the first stage, a posterior instrumentation (T3 to S1), total laminectomy from T11 to S2, L1 pedicle subtraction osteotomy, and correction of the sagittal deformity were planned. In the second stage interbody fusion for all disc levels from T11 to S1 was planned. The apical vertebra was not hypoplastic, so a pedicle subtraction osteotomy was performed successfully. The subtraction osteotomy was similar to the technique in the previous report [5]. After the application of the permanent rods, a dural tear was noticed with the surgical microscope at L3 level due to a very thin dura. The dural tear was repaired successfully using continuous absorbable sutures. The set screws were locked and three transverse connectors were adapted to the instrumentation. The wound was closed meticulously with two passive nonvacuum drains. The patient was taken to the ICU for postoperative management. The operative time of the first stage was 10 hours. According to the anesthesia report total blood loss was 1600 cc, and 4 units of erythrocyte suspension, 4 units of fresh frozen plasma, and 250 cc of cell-saver blood were transfused. There was significant improvement on the patient's neurological examination after extubation. He began to contract his left quadriceps and powers of all his left-sided muscles improved to 4/5. The touch and pain sensations improved on the left side and returned to almost normal on the right extremity.

**Figure 2:**
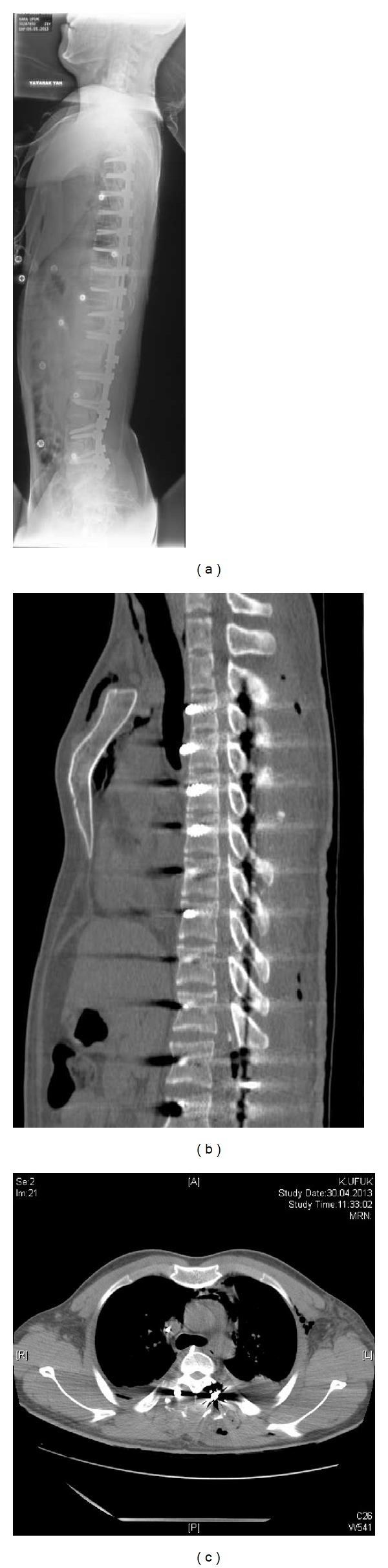
Contusion of the trachea at the carina level by the right T3 pedicle screw.

**Figure 3:**
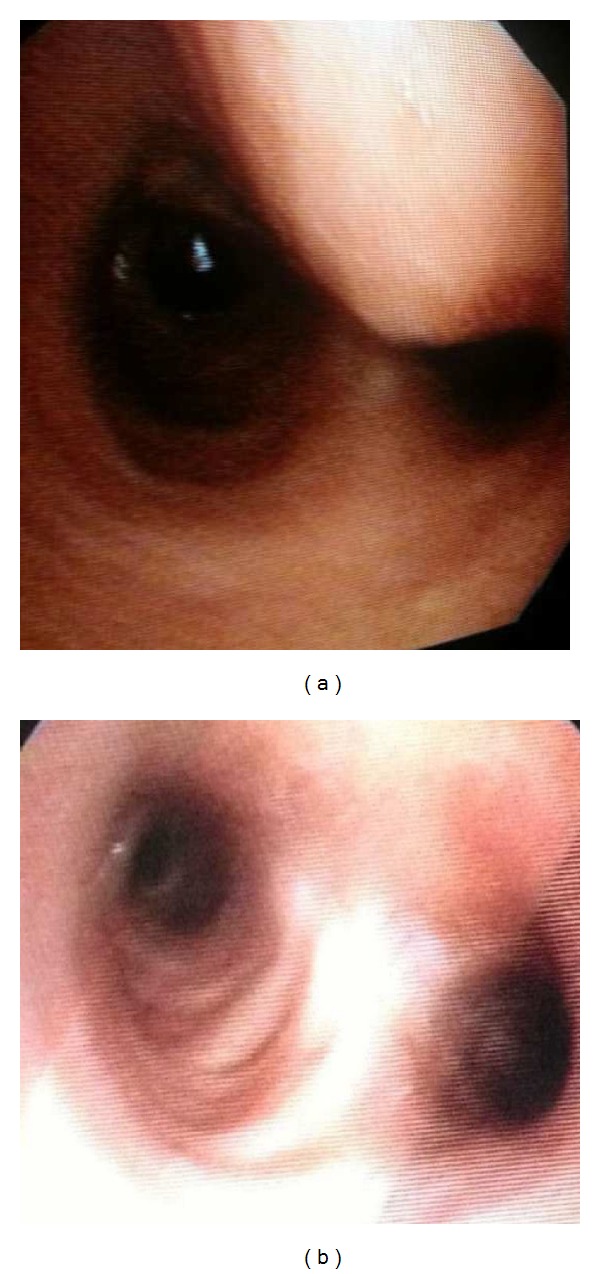
Bronchoscopic view of the trachea at the main carina level. (a) There is an extrinsic pressure on the distal part of trachea and the right main stem bronchus. (b) After the screw was removed and replaced by a 10 mm shorter, the right main bronchus seems open, and there is no endoluminal sign of tracheal tear.

After three days in the ICU the patient was transferred to the floor, allowed to remain only on lateral decubitus positions due to the dural tear. The drains were removed on the third day. On the morning of the fourth day the patient had tachycardia, excessive coughing and respiratory distress with tachypnea. After routine blood tests and thoracic CT, a pneumomediastinum was diagnosed, caused by contusion of the trachea at the carina level by the right T3 pedicle screw (Figures 2(a), 2(b), and 2(c)). A tracheoscopy was done to see the tracheal injury due to the screw (Figures 3(a), and 3(b)). Then the screw was removed and replaced by a 10 mm shorter (30 mm instead of 40) screw. A control tracheoscopy was performed to decide on inserting a tracheal stent. Because the patient's respiratory status improved, the pulmonary specialist decided to follow the patient without a tracheal stent. Tracheal damage was self-limiting so application of an airway stent was not necessary.

The second stage operation was performed 15 days after the first stage. A thoracoabdominal approach was used to replace four-level discs with interbody cages and L1 total corpectomy was also replaced with expandable cage. An anterior approach was made for a two-level interbody fusion in the same stage. After the second stage there were no wound, neurologic, and pulmonary complications. His neurological examination was at the same level after the first stage.

## 3. Discussion

Tracheal tear or laceration is seen very rarely, and almost all cases are iatrogenic. Despite the vast number of intubations and diagnostic or therapeutic endobronchial interventions, iatrogenic tracheal lacerations are rare complications. The reported incidence is approximately 0.005% for orotracheal intubations and 0.05% to 0.19% with double-lumen intubations [6]. Tracheal injuries are uncommon after spinal surgery and instrumentation. In this patient pneumomediastinum occurred because of a pedicle screw that passed through the anterior cortex and injured the trachea. A subcutaneous emphysema alerted on this situation, and it was detected by bronchoscopy (Figure 3(a)). Although there are numerous reports on pedicle screws damaging the big vessels or damaging the spinal cord, there are no reports on pedicle screw injury of the trachea, neither during scoliosis surgery nor during other adult spinal surgeries [7]. Due to the elastic nature of the tracheal wall the damage limited itself and prevented a rupture (Figure 3(b)). Tracheal tears can be observed conservatively or repaired surgically depending on their length. A few cases have healed with endotracheal stents. A tracheal tear can be a fatal complication if missed and not repaired. In this case the tear was small (<3 mm); therefore, it limited itself, and healing was possible with only medical treatment.

## 4. Conclusion

Tracheal tear after spinal surgery is a very uncommon complication. Its management may be challenging, and endoluminal approach is the treatment of choice in the selected cases with small tears. In this report we presented a case with a tracheal tear that developed after spinal instrumentation and was treated conservatively.
